# Genome-Wide Identification and Characterization of Aquaporins and Their Role in the Flower Opening Processes in Carnation (*Dianthus caryophyllus*)

**DOI:** 10.3390/molecules23081895

**Published:** 2018-07-29

**Authors:** Weilong Kong, Mohammed Bendahmane, Xiaopeng Fu

**Affiliations:** 1 Key Laboratory of Horticultural Plant Biology, Ministry of Education, College of Horticulture and Forestry Sciences, Huazhong Agricultural University, Wuhan 430070, China; Asuraprince@126.com (W.K.); mohammed.bendahmane@ens-lyon.fr (M.B.); 2 Key Laboratory of Urban Agriculture in Central China (pilot run), Ministry of Agriculture, Wuhan 430070, China; 3 State Key Laboratory for Hybrid Rice, College of Life Sciences, Wuhan University, Wuhan 430072, China; 4 Laboratoire Reproduction et Development des Plantes, Ecole Normale Supérieure Lyon, 520074 Lyon, France

**Keywords:** AQP, carnation, qRT-PCR, RNA-seq, co-expression network, flower opening process

## Abstract

Aquaporins (AQPs) are associated with the transport of water and other small solutes across biological membranes. Genome-wide identification and characterization will pave the way for further insights into the AQPs’ roles in the commercial carnation (*Dianthus caryophyllus*). This study focuses on the analysis of AQPs in carnation (DcaAQPs) involved in flower opening processes. Thirty DcaAQPs were identified and grouped to five subfamilies: nine PIPs, 11 TIPs, six NIPs, three SIPs, and one XIP. Subsequently, gene structure, protein motifs, and co-expression network of DcaAQPs were analyzed and substrate specificity of DcaAQPs was predicted. qRT-PCR, RNA-seq, and semi-qRTRCR were used for DcaAQP genes expression analysis. The analysis results indicated that DcaAQPs were relatively conserved in gene structure and protein motifs, that DcaAQPs had significant differences in substrate specificity among different subfamilies, and that DcaAQP genes’ expressions were significantly different in roots, stems, leaves and flowers. Five DcaAQP genes (*DcaPIP1;3*, *DcaPIP2;2*, *DcaPIP2;5*, *DcaTIP1;4*, and *DcaTIP2;2*) might play important roles in flower opening process. However, the roles they play are different in flower organs, namely, sepals, petals, stamens, and pistils. Overall, this study provides a theoretical basis for further functional analysis of DcaAQPs.

## 1. Introduction

Aquaporins (AQPs) are integral membrane proteins that belong to the major intrinsic protein (MIP) superfamily [1,2]. AQP1 was first discovered in the erythrocytes of mammals [3]. Since then, hundreds of AQP genes have been identified in eubacteria, archaea, fungi, animals, and plants [2]. A number of diverse AQP isoforms were found in plants: 35 AQPs have been identified in *Arabidopsis thaliana*, 43 in *Zea mays*, 34 in *Oryza sativa*, 58 in *Populus trichocarpa*, 72 in *Glycine max*, 57 in *Brassica rapa* [4,5], 28 in *Beta vulgaris* [6], and so on. AQPs can be classified into seven subfamilies: plasma membrane intrinsic proteins (PIPs), tonoplast intrinsic proteins (TIPs), nodulin 26-like intrinsic protein (NIPs), small basic intrinsic proteins (SIPs), X-intrinsic proteins (XIPs), GlpF-like intrinsic proteins (GIPs), and intrinsic hybrid proteins (HIPs) [2,7].

AQP proteins consist of six trans-membranes (TM1-TM6) connected by five loops (Loops A–Loops E, LA–LE) [8]. The asparagine–proline–alanine (NPA) motifs, aromatic/arginine (ar/R) selectivity filter, and Froger’s positions are related to channel selectivity. The NPA motifs on two half TM helices form the first constrict. The size of this constrict limits the size of the substrates [9]. The second constrict consists of four residues known as ar/R selectivity filter located towards the extracellular side approximately 8 Å away from the NPA regions. The discrepancy in the size and hydrophobicity of the ar/R selectivity filters determines solute transport specificity [10,11]. In addition, Deshmukh et al. provided a precise molecular basis to identify Si accumulators or excluders in higher plants. They reported NIP-III AQPs (Si transporters) with a GSGR selectivity filter and a precise distance of 108 amino acids (AA) between the NPA domains [4].

AQPs play major roles in numerous physiological processes, such as seed germination, reproduction, anther dehiscence, stomatal movement, photosynthesis, petal movement [12,13,14], fruit ripening [2], xylem embolism repair [15], maintenance of cell turgor [16], and cell elongation [17]. AQPs are also involved in the response to environment stresses and in maintaining water homeostasis [18]. In addition, AQPs facilitate the transport of a variety of solutes, such as boron, silicon, ammonia, glycerol, and urea [19].

Carnation (*Dianthus caryophyllus*) is one of the most important cut-flowers, and approximately 200 million carnation plants are sold per year [20]. Carnation flowers wither extremely easily after harvest, seriously affecting their ornamental life. If carnation flower ornamental life could be extended, its economic value would be greatly enhanced. Thus, it is of great importance to find the key genes controlling ornamental life and regulating water balance. AQPs play major roles in plant growth and development, especially in the transport of water and other small solutes as well as water stability under adverse conditions [2,14,19]. Therefore, thousands of studies of AQPs in various species were conducted [2,6]. In carnation, Harada first identified one carnation AQP gene (*DcaPIP1;3*) by suppression subtractive hybridization and found that its expression was induced in the flower opening stages [21]. With the successive release of genome and transcription resources [22,23], Morita reported 26 DcaAQPs based on a simple genome BLAST search, and his study investigated expression of six genes (PIP subfamily) in petals and leaves by qRT-PCR, and examined the expression level of two genes, *DcaPIP2;1* and *DcaPIP1;1*, during the flower opening stages [24]. Limited to a single search approach and genome imperfection, some DcaAQPs were missing and classification errors even occurred in 26 identified DcaAQPs. Here, we performed a genome-wide analysis of AQP gene family members in carnation to examine the expression profile, gene structure, protein motifs, substrate specificity, and co-expression network. The present research will contribute to a better understanding of AQP evolution and functional regulation during flower opening process.

## 2. Results

### 2.1. Identification, Classification, Nomenclature of DcaAQPs, and Properties of DcaAQPs

**Figure 1:**
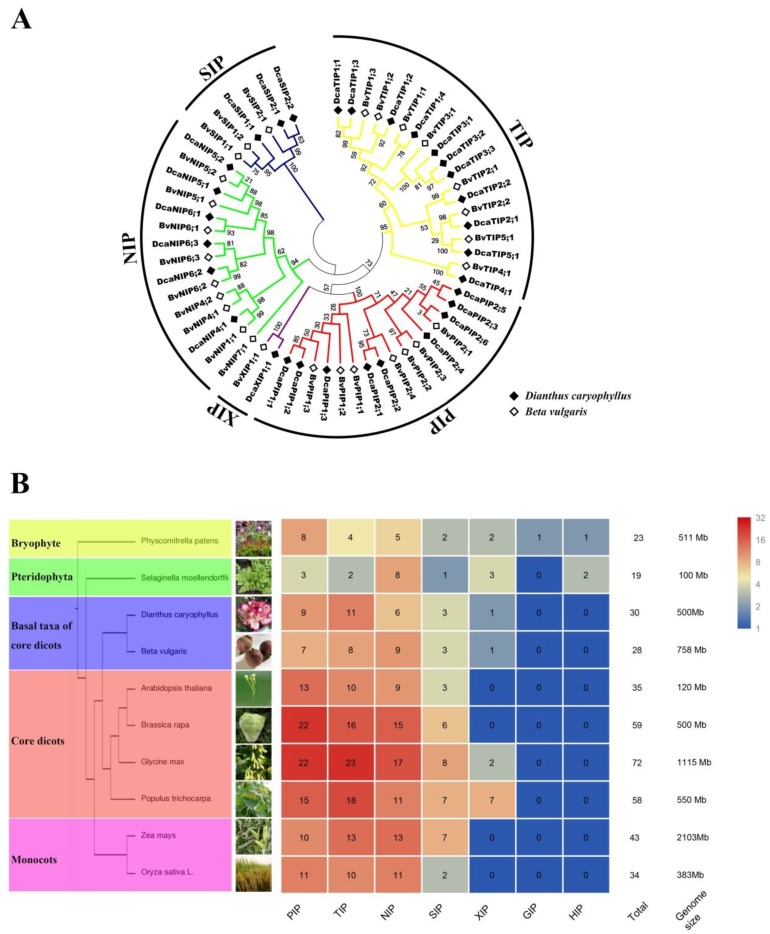
(**A**) Phylogenetic tree from DcaAQPs and *B. vulgaris* L. Multiple alignments were conducted by Clustal W and phylogenetic tree was generated by MEGA 6.0 using Maximum Likelihood (ML) method with 1000 bootstrap replicates. The different subfamilies were indicted by a circle and different colors; (**B**) The distribution of AQPs among different species was compared based on previous report (Deshmukh et al. 2016; Sonah et al. 2017). Schematic of species phylogenetic relationships was built by EvolView (https://www.plob.org/tag/evolview), and the number of AQPs was shown by heat map using R software.

A total of 30 non-redundant sequences were identified as DcaAQPs based on homology search. The sequence of all DcaAQPs ranged from 220–339 amino acids. Of all DcaAQP genes, *DcaPIP2;6*, *DcaTIP2;1*, *DcaNIP4;1*, and *DcaSIP2;1* were found to be incomplete in the genome. Sequences of *DcaPIP2;6*, *DcaTIP2;1*, and *DcaNIP4;1* were obtained by sequencing the PCR amplified full-length genes. The full length sequence of *DcaSIP2;1* was confirmed by transcriptome sequence (*FX318452.1*) [23]. The full length sequences of these four DcaAQPs are provided in Table S1. Besides, in this study, a total of six putative allele ORF pairs were identified with two pairs (*Dca27473.1*/*Dca29035.1* and *Dca44001.1*/*Dca51356.1*) newly detected and never reported by Morita et al. (Table 1) [24]. Based on homologous sequence cluster result with *B. vulgaris*, DcaAQPs were divided into five subfamilies: nine PIPs, 11 TIPs, six NIPs, three SIPs, and one XIP (Figure 1A). We further identified two subgroups of PIPs (three PIP1s and six PIP2s), five subgroups of TIPs (four TIP1s, two TIP2s, three TIP3s, one TIP4 and one TIP5), three subgroups of NIPs (one NIP4, two NIP5s and three NIP6s), two subgroups of SIPs (one SIP1 and two SIP2s) and one subgroup of XIPs (one XIP1). SIPs and XIPs included a small number of DcaAQP genes, while PIPs and TIPs contained a large number (Figure 1A), which is consistent with the findings of previous studies in different species [2,4,5]. In addition, sequence cluster result of carnation with *A. thaliana* and *S. tuberosum* is completely consistent with what Figure 1A and Figure S1 show. Finally, all sequences were named based on sequence cluster results, and most of DcaAQPs nomenclatures in Morita’s paper were adopted. The newly discovered DcaAQPs were named DcaPIP2;6, DcaNIP4;1, DcaNIP6;2, and DcaSIP2;1 (genome ID: Dca21274.1, Dca15023.1, Dca42214.1 and Dca8588.1), respectively. Three DcaAQPs were renamed DcaNIP6;3 (original name DcaNIP5;1), DcaNIP5;1 (original name DcaNIP5;3), and DcaSIP2;2 (original name DcaSIP2;1) (genome ID: Dca25595.1, Dca40630.1 and Dca13275.1), respectively.

The AQP gene family in plants contains a large number of highly divergent proteins [2,4,25]. The investigation of the distribution of AQP genes in plants can elucidate the evolution relationships of AQP gene family. Some representative species reported by previous studies were compared with carnation (Figure 1B), including *Physcomitrella patens*, *Selaginella moellendorffii*, *A. thaliana*, *G. max* L., *Brassica rapa*, *Z. mays* L., *O. sativa* L., *B. vulgaris*, and *P. trichocarpa* [4,7,26] to reveal the distribution difference of AQPs. Furthermore, NIPs’ selection pressure was calculated. The number of AQPs in carnation was lesser than that in higher plants, such as *A. thaliana*, *G. max*, *B. rapa*, *Z. mays* L., *O. sativa* L, and *P. trichocarpa*, which may be attributed to the fact that higher plants have experienced more than once time gene repeat events [27,28], including whole genome duplication (WGD), fragment duplication (FD), and tandem duplication (TD) [29,30,31]. Interestingly, carnation was found to have more AQPs than basal plants such as *P. patens* and *S. moellendorffii*, but both MCScanx and blastP results showed no gene duplication events in carnation. A similar phenomenon was reported in *B. vulgaris*, a closely related species belonging to the Caryophyllales [6]. The result of further subgroup comparison with *P. patens* or *S. moellendorffii* displayed that TIP and NIP subfamilies had more new subgroups such as TIP1-5, NIP1 and NIP4, SIP2 in Caryophyllales plants. To evaluate the evolution pressure of NIP subfamily genes, Ka/Ks ratio was calculated. The results of all Ka/Ks < 1 indicated NIPs with a negative selection (Table S3).

Bioinformatics analysis revealed that molecular weight (MW) of DcaAQPs ranging from 24.45 to 36.71 kDa with an isoelectric point (pI) between 5.04 and 9.62 (Table 1). The majority of AQPs were predicted to have six transmembrane domains (TMDs) except that DcaPIP2;6, DcaTIP1;3, and DcaSIP2;2 had five TMDs, and that DcaTIP1;2, DcaTIP3;3, DcaTIP4;1, and DcaXIP1;1 had seven TMDs. Subcellular localization prediction revealed that all PIPs, NIPs, and XIPs were located on the plasma membrane, while TIPs were present on the vacuole except DcaTIP3;3 which was located on the plasma membrane. DcaSIP1;1 and DcaSIP2;2 were predicted to be located on the plasma membrane, while DcaSIP2;1 on plasma membrane or on vacuoles (Table 1).

### 2.2. Gene Structure and Conserved Motif Analysis of DcaAQPs

**Figure 2:**
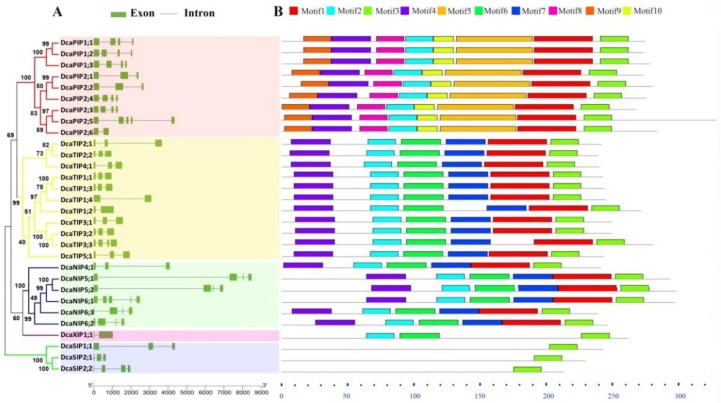
Exon/intron structure (**A**) and motif organization (**B**) of the DcaAQP genes. Multiple alignments were conducted by Clustal W and phylogenetic tree was generated by MEGA 6.0 using Maximum Likelihood (ML) method with 1000 bootstrap replicates. Relative protein or gene lengths can be estimated by gray bars. Exons and introns are represented by green boxes and gray lines, respectively. Information of motifs was provided in Figure S2.

Gene structure and motif organization across five AQP subfamilies were observed in carnation (Figure 2). The observation indicated that PIPs had three or four exons (except *DcaPIP2;5* had five exons) ; TIPs contained two to three exons except that *TIP3;3* had four exons; NIPs contained four to five exons; SIPs had three to four exons; and XIP1;1 had two exons (Figure 2A). Motif 3 was found in all DcaAQPs (Figure 2B and Figure S2), suggesting that the C terminus of AQPs was more conserved than N terminus. Motif 2 was present in all DcaAQPs except SIPs.

The possible reason lies in that Motif 2 represents the NPA domain, but SIPs’ NPA domain was not NPA but NPL or NPT. Motif 1 and Motif 4 specifically existed in PIPs, TIPs, and NIPs subfamilies. Motif 6 was specific to TIPs, NIPs, and XIPs. Motif 7 only existed in the TIPs and NIPs. Motif 5, 8, 9 and 10 were specifically present in the PIPs. Overall, the investigation results of gene structure and protein motif further supported our classification.

### 2.3. Conserved Domain Analysis and Functional Prediction of DcaAQPs

To further understand the possible physiological role and substrate specificity of DcaAQPs, they were aligned and conserved resides (NPA motifs, ar/R selectivity filter and Froger’s position) were analyzed (Table 2) [10,11,32]. The alignment results showed that the NPA motif was more conserved in PIPs and TIPs than in NIPs, SIPs and XIPs, and other types of NPA motifs such as NPS, NPT, NPL, NPV, and NLA found only in NIPs, SIPs and XIPs (Table 2). Different NPA motifs form different first constrict, indicating that unlike PIPs, NIPs, SIPs and XIPs transported the different substrates. Further comparison revealed the significant difference in ar/R selectivity filter and Froger’s positions among the subfamilies (Table 2). Prediction of DcaAQPs functions, based on key protein domains conservation [10,11,33,34,35,36], showed that PIPs were transporter of Boron, CO_2_, H_2_O_2_, Urea, and Ammonia, that TIPs were transporter of H_2_O_2_, Urea, that NIPs were transporters of Boron, and that PIPs and TIPs transported multiple substrates and water, which were important for the growth and development of plants (Table 2).

### 2.4. Co-Expression Network

**Figure 3:**
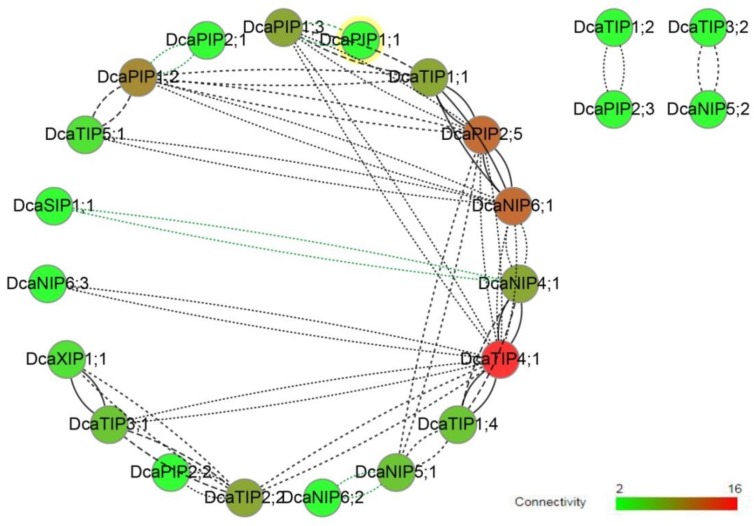
Co-expression network of DcaAQP genes constructed based on the RNA-seq results in this study. The network was obtained with CoExpNetViz (Tzfadia et al. 2016) and visualized by Cytoscape V. 3.1.0 (Shannon et al. 2003). Black and green lines denote positive correlation and negative correlation, respectively. Correlation from weak to strong represents dotted line to solid line. Connectivity from weak to strong represents from green to red.

The results of co-expression analysis revealed that there was a positive correlation between most of DcaAQPs (Figure 3), that a high connectivity and high correlation were found among DcaTIP4;1, DcaNIP6;1, DcaPIP2;5, and DcaNIP4;1, that a medium connectivity was found among DcsPIP1;2, DcaPIP1;3, DcaTIP1;1, and DcaPIP2;2. Interestingly, four pairs of negative correlation were found, namely, DcaPIP1;1 and DcaPIP1;3, DcaPIP1;2 and DcaPIP2;1, DcaSIP1;1 and DcaNIP4;1, and DcaNIP5;1 and DcaNIP6;2. PIPs and TIPs were reported to be the main transporters, and their transport capacity was much stronger than NIPs in previous studies [2,19,37]. This study revealed that DcaNIP6;1 and DcaNIP4;1 had high connectivity with TIPs and PIPs, which suggested that they may play an important role in assisting PIPs and TIPs in transporting substrates.

### 2.5. Expression of DcaAQP Genes in Different Tissues

**Figure 4:**
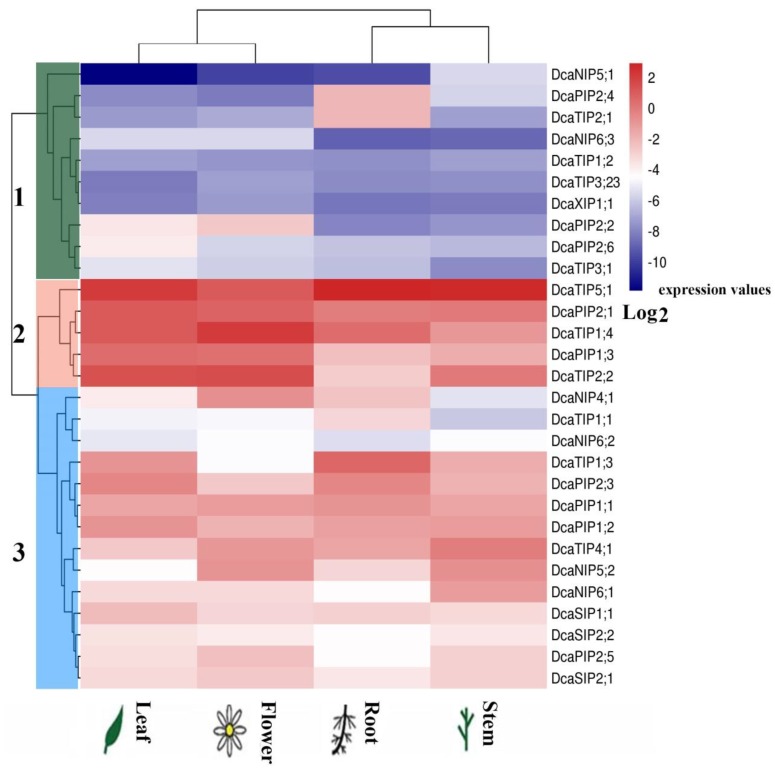
The qRT-PCR analysis of expression profiles in different tissues. Expression values of DcaAQP genes were calculated by comparative CT(2^−ΔΔCT^) method. A heat-map shows the hierarchical clustering of relative expression of all DcaAQP genes across in four different tissues analyzed. The vertical color scale at right of the image represents log_2_^expression values^: red indicates a high level and blue represents a low of transcript abundance.

To explore the function of DcaAQPs, the expression levels of all DcaAQP genes were analyzed by qRT-PCR. The analysis results showed that DcaAQP genes were expressed in all tissues, but exhibited different relative expression levels in different tissues (Figure 4). Of all DcaAQP genes, PIP and TIP subfamily genes showed higher expression levels than NIP, SIP and XIP subfamily genes. Based on the expression levels, all DcaAQP genes clustered into three major groups (Figure 4). Group 1 contained 10 genes (*DcaPIP2;2*, *DcaPIP2;4*, *DcaPIP2;6*, *DcaTIP1;2*, *DcaTIP2;1*, *DcaTIP3;1*, *DcaTIP3;23*, *DcaNIP5;1*, *DcaNIP6;3*, and *DcaXIP1;1*) with relatively low expression level in all tissues or relatively high expression in only one tissue. For example, *DcaPIP2;2* had relatively high expression only in flowers, and *DcaPIP2;4* and *DcaTIP2;1* only in roots. Group 2 contained five genes, *DcaTIP5;1*, *DcaPIP2;1*, *DcaTIP1;4*, *DcaPIP1;3*, and *DcaTIP2;2* with high expression levels in all tissues. These five DcaAQP genes played a major role during the entire process of plant growth and development including flower opening process. Group 3 contained 14 genes (*DcaPIP1;1*, *DcaPIP1;2*, *DcaPIP2;3*, *DcaPIP2;5*, *DcaTIP1;1*, *DcaTIP1;3*, *DcaTIP4;1*, *DcaNIP4;1*, *DcaNIP5;2*, *DcaNIP6;1*, *DcaNIP6;2*, *DcaSIP1;1*, *DcaSIP2;1*, and *DcaSIP2;2*). All 14 of these DcaAQP genes showed a diverse pattern of expression. For example, *DcaNIP4;1* was highly expressed in flowers and roots. *DcaTIP1;3* and *DcaPIP2;3* were highly expressed in roots and leaves. *DcaTIP4;1* and *DcaNIP5;2* were highly expressed in flowers and stems. *DcaNIP6;1* showed higher expression in stems than in other tissues.

### 2.6. Expression Patterns of DcaAQP Genes with RNA-Seq and Semi-qRTPCR

**Figure 5:**
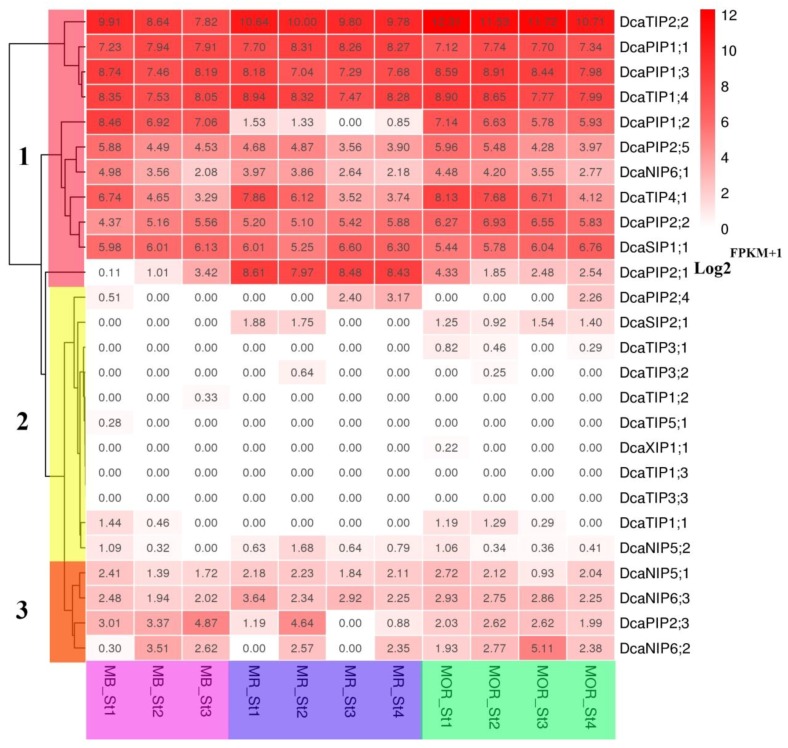
Expression profiles of DcaAQP genes during flower opening stages. MB, MR and MOR represent three carnation cultivars. St1 means stage1, St2 means stage2, St3 means stage3, St4 means stage4. A heat-map shows the hierarchical clustering of relative expression of all DcaAQP genes during flower opening stages analyzed. The vertical color scale at right of the image represents log_2_^FPKM+1^: the expression level were displayed by the depth of red.

The expression patterns of all DcaAQP genes during flower opening stages were examined. Based on the expression levels, AQP genes clustered into three major groups (Figure 5, FRKM results in Table S4). The genes in Group 2 (including 11 members) showed low expression level in all stages, specially *DcaTIP1;3* and *DcaTIP3;3*. Group 3 (including four members) showed medium expression level in most stages. Since AQP is functional protein rather than transcriptional factor, we are more concerned about high expression level of DcaAQP genes, and Group 1 (including11 members) showed high expression in most stages. For example, *DcaTIP2;2*, *DcaPIP1;1*, *DcaPIP1;3*, and *DcaTIP1;4* in Group 1 maintained high expression level in all stages. In Group 1, *DcaPIP2;5*, *DcaNIP6;1*, and *DcaTIP4;1* showed a downward expression trend, while *DcaPIP2;2* and *DcaSIP1;1* showed a upward expression trend. Interestingly, *DcaPIP1;2* and *DcaPIP2;1* showed species-specific expression patterns and their expression patterns were the opposite. *DcaPIP1;2* exhibited a high expression level during all the stages in MB and MOR, but low expression level in MR. However, *DcaPIP2;1* exhibited low expression level during all the stages in MB and MOR, but high expression level in MR. Based on the facts that PIP and TIP have high water permeability and that these genes exhibited high expression, it could be speculated that these DcaAQPs transport adequate water to help the flower opening and petal cell expansion. High-expression *DcaNIP6;1* could transport boron during flower opening stages and high-expression *DcaTIP4;1* and *DcaSIP1;1* could transport unknown substrates or assist PIP and TIP in transporting water.

**Figure 6:**
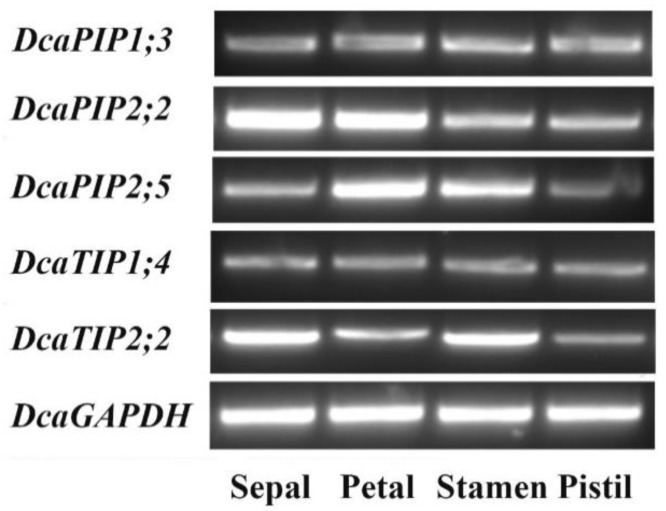
Expression profile of DcaAQP genes in different flower organs. Expression profile was examined by semi-qRT-PCR. *DcaGAPDH* was used as standard control to normalized the data.

Given TIP and PIP high water transport efficiency and substrate diversity, high-expression TIP and PIP genes were focused on during flowering stages. Since *DcaPIP1;1* and *DcaPIP2;1* in different organs have already been reported [24], and *DcaPIP1;2* and *DcaPIP2;1* were species-specific and were not representative, these four genes were not taken into consideration in the further study (Figure 5). Therefore, this study further examined the expression patterns of five TIP and PIP genes, namely, *DcaPIP1;3*, *DcaPIP2;2*, *DcaPIP2;5*, *DcaTIP1;4*, and *DcaTIP2;2* in various flower organs (Figure 6). The results indicated that *DcaPIP1;3* and *DcaTIP1;4* showed a constitutive expression patterns, that *DcaPIP2;2* had advantage expression in sepal and petal, that *DcaPIP2;5* had advantage expression in petal and stamen, and that *DcaTIP2;2* showed advantage expression in sepal and stamen.

## 3. Discussion

With the constantly released plant genomes, an increasing number of family genes have been identified in numerous plants [1,2,19,38,39,40,41]. However, some family genes are missing in the previous reports due to single identification method or imperfect genome. In order to get reliable results, multiple identification and verification methods should be adopted. In this study, we could conduct the mutual correction by different genomic versions [6], the correction by transcription assembly results, and the correction by homology analysis of sequences from same family or genera species. We could also adopt multiple homology search methods and sequencing verification [6]. Owing to the adoption of multiple methods, this study discovered four new DcaAQPs and two new allele ORFs relative to previous reports [24].

Few literatures have reported the AQPs of the plants from angiosperms to the core dicots. Since carnation belongs to Caryophyllales, which lies on basal taxa of core dicots, whole genome identification of AQPs in carnation could provide the insight into the evolution of AQPs. As lower plants, *P. patens* and *S. moellendorffii* have completed the differentiation from PIP subfamily to the PIP1 and PIP2 subgroups, but TIP subfamily has not differentiated, therefore it only has TIP6 subgroup (Figure 1). In this study, TIP subfamily was found to have TIP1-TIP5 subgroups in carnation. Our findings confirmed Maurel’s hypothesis that the subdivision of TIPs was later than that of PIPs [2]. Based on it, it can be deduced that the subdivision of TIPs happened in the evolution process from gymnosperms to basal taxa of core dicots. In addition, the emergence of new NIP4 subgroups and SIP2 subgroups led to the increase in the number of AQPs in Caryophyllales plants relative to lower plants, suggesting that the new subgroup may be related to the adaptation of plants to a changing environment. Our results also confirmed that the loss of HIPs and GIPs occurred in the evolution process from ancient species to basal taxa of core dicots, and that the loss of GIPs was earlier than that of HIPs [2,4,5]. However, XIPs has been preserved in Caryophyllales, and its loss in some higher plants occurred after divergence of the basal core dicot and core dicot. It should be noted that Si (silicon) has many beneficial effects for plants [4,25,42]. Recently, Soundararaja et al. reported that the exogenous supplementation of Si improved the recovery of hyperhydric shoots in carnation [43]. But it was reported that there was a lack of NIP-III AQPs transporting Si, Ge, As, and B in some plant species. Carnation is a Si non-accumulator [43] and it doesn’t have NIP-III AQP either, which is similar to other Si non-accumulator higher plants, such as Solanaceae plants, *Arabidopsis*, and so on [4,5]. Interestingly, one Si-efflux transporter (Dca16341.1) was identified in carnation (Figure S3). Our results supported the findings of Deshmukh et al. and Sonash et al. that the presence of Si permeable NIP-IIIs is the critical factor in determining the ability of a plant to absorb Si [4,5].

This study finds that the same subfamilies have similar gene structure and the conserved elements, but their expression patterns are very different (Figure 2, Figure 4 and Figure 5), indicating that functional differentiation occurring in different members of the same subfamily plays important roles in the adaptation of plants to change environments. The results of expression analysis showed that the transcripts of PIP and TIP subfamily members are highly abundant in all examined tissues and all flower opening stages, which is consistent with the observation in other plant species such as maize, *Arabidopsis*, tomato and potato [2,32,44,45]. Considering that PIPs and TIPs are highly permeable to water [14,38,46], their high abundance indicated their crucial roles in intracellular, cellular, organic, and whole plant water balance in carnation. *DcaNIP4;1* and *DcaPIP2;2* had advantage expressions in flowers, indicating their important roles in flower opening [21,47]. *DcaTIP1;3* and *DcaPIP2;3* had advantage expression in roots and leaves. *DcaPIP2;4* and *DcaTIP2;1* also had advantage expression in roots, indicating they play key role in leaf hydraulic conductance, petal expansion, and water absorption. *DcaNIP5;2* was advantageously expressed in flowers and stems, indicating *DcaNIP5;2* play a key role in water long distance transport.

Morita reported that *DcaPIP1;1* and *DcaPIP1;3* had a higher expression level in flowers than in leaves, and that *DcaPIP1;1* showed a higher expression level than *DcaPIP1;3*. Thus, *DcaPIP1;1* was examined in flower opening stages [24]. Our results also showed these two DcaAQP genes had higher expression in flowers than in leaves, but *DcaPIP1;3* was higher expressed than *DcaPIP1;1*. Interestingly, the expression patterns of these two DcaAQP genes were similar during flower opening stages and were clustered together (Figure 4 and Figure 5). Harada reported that *DcaPIP1;3* was involved in petal cell expansion [21], indicating that *DcaPIP1;1* might also be involved in petal cell expansion. Morita reported that *DcaPIP2;1* and *DcaPIP2;2* had a higher expression level in flowers than in leaves, and that *DcaPIP1;1* showed a higher expression level than *DcaPIP1;3*, thus *DcaPIP1;1* was examined in flower opening stages [24]. Our results also showed that the same expression patterns were found in flowers and in leaves, which agrees with Morita’s finding. However, our study finds that *DcaPIP2;2* plays a greater constitutive role than *DcaPIP2;1* (Figure 4 and Figure 5). In rose petals, the role of *Rh-PIP2;1*, the homologous gene *of DcaPIP2;2*, in regulating petal cell expansion was confirmed, suggesting that *DcaPIP2;2* could be a major gene regulating petal cell expansion. Besides, *DcaTIP2;2* and *DcaTIP1;4* showed similar high expression pattern during flower opening stages and both of them had advantage expression in flowers, indicating that *DcaTIP2;2* and *DcaTIP1;4* have same function in flowers and they worked together to maintain a large amount of water supply during the flower opening stages. Similarly, RhTIP1;1(the homology protein of DcaTIP1;4) was reported to be involved in the water transport during the flower opening of cut roses [48]. However, this study found that *DcaTIP1;4* had a relatively high expression in roots, and that *DcaTIP2;2* had relatively high expression in leaves and stems, indicating these two genes are functionally different in leaves, roots, and stems. This study also found that *DcaNIP6;1* and *DcaTIP4;1* showed downward trend during flower opening stages and that they showed advantage expression in stems, indicating that *DcaNIP6;1* and *DcaTIP4;1* could play an important role in water/nutrient long distance transportation and water supply in flower opening early stage. *DcaSIP1;1* showed an upward trend, suggesting it could play a role in flower opening late stage. Previous studies have reported that dimers can greatly increase the transport capacity [49,50]. Yaneff reported that PIP1 and PIP2 heterotetramers had strong water transport activity [51,52]. *Xenopus* oocyte experiments also demonstrated that PIP1 and PIP2 heterotetramers enhanced the water permeability of PIP1 which originally had no water permeability [51,53,54,55]. This study found that high positive correlation was found among PIP1s and PIP2s. It can be deduced that PIP1s and PIP2s can form a heterodimer or heterotetramers in carnation.

Previous studies suggested TIP3s’ role in flowering and seed development, specifically in desiccation process. TIP3s genes showed high expression in seeds or flowers in *Arabidopsis* [56], castor bean [57], canola [5], flax [58], and sugar beet [6]. But in this study, TIP3s genes showed low expression in all tissues and in all flower opening stages. Similar findings were reported in *Jatropha curcas* L. [38] and banana [59]. These results suggested that TIP3s gene function is not strictly conserved in plants, which TIP3s genes don’t play a role in flowers or seeds in some species and that TIP3s function may be replaced by other AQP proteins. For example, *DcaTIP4;1* showed advantages expression in flowers and high expression during all flowering stages. These results indicated that the functions of the same subgroup are differentiated in different species, and that the aquaporin family may be functionally redundant.

Further analysis found that DcaAQPs with high expression level in flowers were differently expressed in various flower organs (Figure 5). Based on these, it could be concluded that *DcaPIP1;3* and *DcaPIP1;4* might play a constitutive role in all flowers, that *DcaPIP2;2* might play a main role in sepal and petal and it could be very important for flower morphology maintenance, that *DcaPIP2;5* might play a main role in sepal and could be involved in petal cell expansion and that *DcaTIP2;2* could play a role in stamen elongation. Tissue-specific expression were reported in the ice plant, *J. curcas*, and *Hevea brasiliensis* [6,38,54,60].

## 4. Materials and Methods

### 4.1. Plant Materials, RNA and DNA Extraction

Plants were grown in growth chamber at 25 °C with 60% relative humidity and a light regime of 14 h/10 h day/night. The plants were watered twice a week and were fertilized once a week. Tissues from roots, stems, leaves and flowers from the cultivar ‘Mini-Pink’, and individual flower organs such as sepals, petals, stamens and pistils of the cultivar ‘Master’ were collected, frozen in liquid nitrogen immediately and stored at −70 °C. Total RNA was extracted using EASYspin Plus plant RNA kit (AidLab, Beijing, China) and RNA was reverse transcribed into cDNA using the PrimeScript RT reagent Kit (TakaRa, Dalian, China). The reverse transcription of cDNA was diluted 10-fold for qRT-PCR.

### 4.2. Identification of DcaAQPs

DcaAQPs were identified by the Hidden Markov model (HMM) and BLAST homology searches [6]. The carnation proteome was downloaded from Carnation DB (http://carnation.kazusa.or.jp/index.html). The HMM of the MIP domain (PF00230) was downloaded from the Sanger database (http://pfam.xfam.org/family/PF00230), and PF00230 was used to query carnation proteome using HMMER 3.0 software (http://hmmer.org/). 35 AtAQPs were downloaded from TAIR Database (https://www.arabidopsis.org/browse/genefamily/Aquaporins.jsp) and used to search DcaAQPs with BLASTP tools in Carnation DB with cut-off E-value of e-5. All the non-redundant gene sequences were analyzed by SMART (http://smart.embl-heidelberg.de/) [61] and Pfam (http://pfam.xfam.org/search/sequence) [62]. Sequences encoding complete MIP domain and two NPA motifs were considered as putative AQP genes [63]. In order to prevent genome assembly and annotation errors, primers were designed for cloning the sequences with partial transmembrane structures lost. Randomly selected nine AQPs were verified for the sequence correctness by sequencing the PCR amplified full-length genes (Primers in Table S2). Similarly, homologs of Si-efflux transporter were identified in carnation using BLASTp search with known efflux transporters sequences [5,64,65,66].

### 4.3. Phylogenetic Analysis and Gene Duplication of DcaAQPs

Multiple alignments with other plants species (*A. thaliana*, *S. tuberosum*) were conducted by Clustal W and phylogenetic dendrogram was generated by MEGA 6.0 using the Maximum Likelihood (ML) method with 1000 bootstrap replicates [67,68,69]. In order to obtain correct classification, the same Caryophyllales species *Beta vulgaris* L. also was used for phylogenetic analysis with carnation [6]. Subsequently, DcaAQPs were systematically named based on the clustering results [38]. At last, gene duplication patterns of DcaAQPs were analyzed by MCScanX and BLASTP (1.0E-10, identity > 80%) [70].

### 4.4. Bioinformatics Analysis of DcaAQPs

Information of introns and exons for DcaAQP genes was obtained from Carnation DB, Exon-intron structure was analyzed by GSDS 2.0 (http://gsds.cbi.pku.edu.cn/) [71] using default parameters. Conserved motifs were generated by MEME suit (http://meme-suite.org/tools/meme) [72], and the following parameter settings were used: the number of motifs was set as 10, the optimum width of motifs was 3 to 60, and other parameters were set as default values [40,63]. The transmembrane domain prediction was studied using TMHMM Server v.2.0 (http://www.cbs.dtu.dk/services/TMHMM/). The MW and pI of the amino acid sequences were predicted using online program ProtParam (http://web.expasy.org/protparam/). Subcellular localization was analyzed by Plant-mPloc server (http://www.csbio.sjtu.edu.cn/bioinf/plant-multi/) [40].

### 4.5. Ka/Ks Analysis and Co-Expression Network

Non-synonymous (Ka) and synonymous (Ks) substitution ratio of NIP genes was calculated to test selection pressure. The alignments generated by ClustalW and the corresponding cDNA sequences were submitted to the online program PAL2NAL (http://www.bork.embl.de/pal2nal/) [73], which automatically calculates Ks and Ka by the codeml program in PAML [74]. RNA-seq datasets were used to construct co-expression network in Comparative Co-Expression Network Construction and Visualization tool (CoExpNetViz) [75] with the following Parameters: Pearson product-moment correlation coefficient (Pearson r), correlation thresholds with lower percentile rank 5 and upper percentile rank 95 considered as significant association, and the entire list of DcaAQPs was used as bit genes [5]. The network was visualized using Cytoscape V. 3.1.0, the correlation coefficient >0.75 or <−0.75 was adopted [76].

### 4.6. qRT-PCR Analysis in Different Tissues

Primers were designed by Primer 5.0 in specific regions or 3′-/5′-UTR regions (Primers in file2). Genes with high similarity (*DcaTIP3;2* and *DcaTIP3;3*) were seen as identical genes in qRT-PCR analysis. qRT-PCR reaction (10 μL) was formulated using SYBR^®^ Premix Ex TaqTM kit (TaKaRa, Dalian, China). qRT-PCR was carried out in 384-well plates on ABI Q7 Real-Time PCR System (ABI, Carlsbad, CA, USA) and melt curves were generated to check the amplification specificity. *DcaGADPH* (glyceraldehyde-3-phosphate dehydrogenase) was used as an internal reference gene to normalize expression [77]. Three biological and technical replicates were carried out for each qRT-PCR accession. The cycling parameters as follow: heating for 4 min at 95 °C, 40 cycles of denaturation at 95 °C for 10 s, annealing for 20 s at 60 °C, and extension at 72 °C for 35 s. The comparative CT(2^−ΔΔCT^) method was used to calculate the relative quantitation of AQP genes and heat map was created by R software [38,40,78].

### 4.7. RNA-Seq Analysis during Flower Opening Stages and Semi-qRT-PCR in Individual Flower Organs

RNA-seq data (DRX118351-DRX118354; DRX091738-DRX091740; DRX091742-DRX091745) were collected from NCBI and used to analyze the expression profiles of DcaAQP genes for three carnation cultivars (MOR, MB and MR) during flower opening stages. Tophat2 was used to map with reference genome, and Cufflinks was used to calculate gene expression [79]. The fragment per kilobase of exon per million fragments mapped (FPKM) method was adopted for the determination of transcript levels, and heat map was generated based on the log_2_^FPKM+1^ values for each gene. Furthermore, high expression DcaAQP genes in all flowers opening stages were detected in sepals, petals, stamens and pistils by semi-qRTPCR. *DcaGAPDH* served as an internal reference gene in semi-qRTPCR analysis (Primers in Table S2) [77,80] with the reaction procedures: heating at 95 °C for 3 min, denaturation at 94 °C for 30 s, annealing at 60 °C for 30 s, extension at 72 °C for 40 s, 24 to 30 cycles, at last extension at 72 °C for 10 min [81].

## 5. Conclusions

Genome-wide identification and analysis of AQPs in carnation highlighted several novel findings explaining AQP evolution and functional regulation during flower opening process. In this study, 30 DcaAQPs were identified, including nine PIPs, 11 TIPs, six NIPs, three SIPs and one XIP. Compared to lower plants *P. patens* and *S. moellendorffii*, carnation has more AQPs, which was mainly attributed to the new subgroups, such as NIP1s, NIP4s, and TIP1s-TIP5s. Our results support Maurel’s hypothesis that the subdivision of TIPs is later to that of PIPs. It could be speculated that the subdivision of TIPs happened in the evolution process from gymnosperms to basal taxa of core dicots. No NIPIII AQP was found in carnation and sugar beet. One Si-efflux transporter was identified firstly in carnation, and similar findings was reported in Brassicaceae species [5]. But XIPs was found to exist in Caryophyllales plants, suggesting XIPs losses might have occurred after the differentiation of basal taxa of core dicots. Expression profiles suggested that DcaAQP genes exhibited different expression patterns during flower opening process and in different tissues. Five PIPs and TIPs were found to exhibit high expression levels during all flower opening stages, but they were differently expressed in four flower organs. As flower-opening-related candidate genes, *DcaPIP1;3*, *DcaPIP2;2*, *DcaPIP2;5*, *DcaTIP1;4*, and *DcaTIP2;2* remain to be further explored. NIPs have high connectivity and high correction with TIPs and PIPs, suggesting that NIPs may play an important role in assisting PIPs and TIPs in water and solute transport during the flower opening process. TIP3 showed the expression patterns different from previous reports [5,6,56,57,58], and TIP4s might play a greater role than TIP3s in flower development. *TIP4;1* was found to be advantageously expressed in flower tissues and maintained a high expression level in all flower opening process. These results provide an insight into the biological roles of individual DcaAQPs in carnation and this information can be applied to explore future applications for prolonging cut-flowers ornamental life.

## Figures and Tables

**Table 1:** Nomenclature and protein properties of DcaAQPs.

Subfamily	Gene	Accession ID	AA Length	MW (kDa)	pI	TMD	Subcellular Localization	Putative Allele ORF
PIP	*DcaPIP1;1*	Dca52692.1	285	30.4083	8.86	6	plas	
	*DcaPIP1;2*	Dca27473.1	284	30.3473	8.84	6	plas	Dca29035.1
	* *DcaPIP1;3*	Dca31714.1	289	30.857	9.14	6	plas	
	* *DcaPIP2;1*	Dca59969.1	284	30.4886	9.23	6	plas	Dca47122.1
	* *DcaPIP2;2*	Dca36456.1	291	31.2254	9.2	6	plas	
	* *DcaPIP2;3*	Dca37662.1	278	29.6184	8.58	6	plas	Dca51181.1
	* *DcaPIP2;4*	Dca7764.1	286	30.7608	8.58	6	plas	Dca23336.1
	* *DcaPIP2;5*	Dca12710.1	339	36.7057	9.57	6	plas	
	* *DcaPIP2;6*	Dca21274.1	295	31.586	9.21	5	plas	
TIP	*DcaTIP1;1*	Dca5708.1	251	25.7188	5.36	6	vacu	
	*DcaTIP1;2*	Dca9296.1	281	30.0387	5.11	7	vacu	
	* *DcaTIP1;3*	Dca44001.1	252	25.9331	5.43	5	vacu	Dca51356.1
	* *DcaTIP1;4*	Dca50626.1	253	25.9171	5.04	6	vacu	
	* *DcaTIP2;1*	Dca15468.1	250	25.3706	5.33	6	vacu	
	* *DcaTIP2;2*	Dca20683.1	247	25.1731	5.76	6	vacu	
	*DcaTIP3;1*	Dca4416.1	258	27.2657	6.7	6	vacu	Dca20060.1
	*DcaTIP3;2*	Dca4417.1	258	27.2918	7.15	6	vacu	
	*DcaTIP3;3*	Dca20061.1	290	30.7138	7.15	7	plas	
	*DcaTIP4;1*	Dca14656.1	248	25.8422	5.34	7	vacu	
	*DcaTIP5;1*	Dca14871.1	252	26.2813	6.39	6	vacu	
NIP	* *DcaNIP4;1*	Dca15023.1	250	26.7095	9.35	6	plas	
	*DcaNIP5;1*	Dca40630.1	304	31.5687	8.38	6	plas	
	*DcaNIP5;2*	Dca29915.1	308	31.7267	8.75	6	plas	
	*DcaNIP6;1*	Dca29994.1	307	31.8083	6.42	6	plas	
	*DcaNIP6;2*	Dca42214.1	247	25.8832	8.76	6	plas	
	*DcaNIP6;3*	Dca25595.1	254	26.2389	6.05	6	plas	
SIP	*DcaSIP1;1*	Dca30655.1	251	26.3213	9.62	6	plas	
	* *DcaSIP2;1*	Dca8588.1	271	26.0338	8.41	6	vacu/plas	
	*DcaSIP2;2*	Dca13275.1	237	24.448	9.54	5	plas	
XIP	*DcaXIP1;1*	Dca56078.1	220	28.9014	9.05	7	plas	

Plas: plasma membrane; Vacu: vacuolar membrane; AA: Amino acid; MW: molecular weight; pI: isoelectric point; TMD: transmembrane domain; ORF: opening reading frame. * Sequence were randomly selected for verification. The MW and pI of the amino acid sequences were predicted using online program ProtParam (http://web.expasy.org/protparam/). The TMD prediction was studied using TMHMM Server v.2.0 (http://www.cbs.dtu.dk/services/TMHMM/). Subcellular localization was analyzed by Plant-mPloc server (http://www.csbio.sjtu.edu.cn/bioinf/plant-multi/).

**Table 2:** Amino acid composition of the NPA motifs, ar/R selectivity filter and Froger’s positions of DcaAQPs.

Gene	NPA		Ar/R Selectivity Filter	Froger’s Position	Hove et al.	Azad et al.
	LB	LE	H2,H5,LE1,LE2	P1,P2,P3,P4,P5		
DcaPIP1;1	SGGHINPAVT	GTGINPARSLG	F,H,T,R	Q,S,A,F,W	Boron,CO_2_,H_2_O_2_,Urea	CO_2_,H_2_O_2_
DcaPIP1;2	SGGHINPAVT	GTGINPARSLG	F,H,T,R	Q,S,A,F,W	Boron,CO_2_,H_2_O_2_,Urea	CO_2_,H_2_O_2_
DcaPIP1;3	SGGHINPAVT	GTGINPARSLG	F,H,T,R	Q,S,A,F,W	Boron,CO_2_,H_2_O_2_,Urea	CO_2_,H_2_O_2_
DcaPIP2;1	SGGHINPAVT	GTGINPARSFG	F,H,T,R	M,S,A,F,W	Ammonia,Urea	Ammonia
DcaPIP2;2	SGGHINPAVT	GTGINPARSFG	F,H,T,R	M,S,A,F,W	Ammonia,Urea	Ammonia
DcaPIP2;3	SGGHINPAVT	GTGINPARSFG	F,H,T,R	Q,S,A,F,W	H_2_O_2_,Urea	H_2_O_2_
DcaPIP2;4	SGGHINPAVT	GTGINPARSFG	F,H,T,R	Q,S,A,F,W	H_2_O_2_,Urea	H_2_O_2_
DcaPIP2;5	SGGHINPAVT	GTGINPARSFG	F,H,T,R	Q,S,A,F,W	H_2_O_2_,Urea	H_2_O_2_
DcaPIP2;6	SGGHINPAVT	GTGINPARSLG	F,H,T,R	Q,S,A,F,W	Boron,CO_2_,H_2_O_2_,Urea	CO_2_
DcaTIP1;1	SGGHVNPAIT	GASMNPAVSFG	H,I,A,V	T,S,A,Y,W		
DcaTIP1;2	SGGHVNPAVT	GASMNPAVTFG	H,I,A,V	T,T,A,Y,W	Urea	
DcaTIP1;3	SGGHVNPAIT	GASMNPAVSFG	H,I,A,V	T,S,A,Y,W		
DcaTIP1;4	SGGHVNPAVT	GASMNPAVSFG	H,I,A,V	T,S,A,Y,W	H_2_O_2_,Urea	H_2_O_2_,Urea
DcaTIP2;1	SGGHLNPAVT	GGSMNPARSFG	H,I,G,R	T,S,A,Y,W	H_2_O_2_,Urea	H_2_O_2_,Urea
DcaTIP2;2	SGGHVNPAVT	GGSMNPARSFG	H,I,G,R	T,S,A,Y,W	H_2_O_2_,Urea	H_2_O_2_,Urea
DcaTIP3;1	SGGHVNPAVT	GASMNPARAFG	H,I,A,R	T,A,A,Y,W	H_2_O_2_,Urea	
DcaTIP3;2	SGGHVNPAVT	GASMNPARAFG	H,I,A,R	T,A,A,Y,W	H_2_O_2_,Urea	
DcaTIP3;3	SGGHVNPAVT	GASMNPARAFG	H,I,A,R	T,A,A,Y,W	H_2_O_2_,Urea	
DcaTIP4;1	SGGHLNPAVT	AASMNPARSFG	H,I,A,R	T,S,A,Y,W		
DcaTIP5;1	SGGHVNPAVT	GGSMNPAYSFG	N,V,G,Y	T,S,A,Y,W		
DcaNIP4;1	SGAHFNPAVT	GASMNPARSIG	W,F,A,R	F,S,A,Y,I		
DcaNIP5;1	SGAHLNPSLT	GGSMNPVRTLG	A,I,G,R	F,T,A,Y,L	Boron	Boron
DcaNIP5;2	SGAHLNPSLT	GGSMNPVRTLG	A,I,G,R	F,T,A,Y,L	Boron	Boron
DcaNIP6;1	SGAHLNPALT	GASMNPVRTLG	A,I,A,R	F,T,A,Y,L	Boron	
DcaNIP6;2	SGAHINLAVS	GGSMNPARTVG	S,I,G,R	Y,T,A,Y,I		
DcaNIP6;3	SKAHFNPAVT	GASMNPARTLG	S,I,A,R	Y,T,A,Y,M		
DcaSIP1;1	GGASFNPTGT	GFSMNPANAFG	I,V,P,N	M,A,A,Y,W		
DcaSIP2;1	KAGNYNPLTL	GGCMNPASVMG	S,Q,G,S	F,V,A,Y,W		
DcaSIP2;2	NGAAYNPLTV	GGCMNPASVMG	S,H,G,S	F,V,A,Y,W		
DcaXIP1;1	SGGHINPSVT	GAGMNPARCVG	I,T,A,R	V,C,A,Y,W		

## Supplementary Materials

Supplementary materials are available on line.

## Abbreviations

AQPs	Aquaporins
DcaAQPs	AQPs in D. caryophyllus
PIPs	Plasma membrane intrinsic proteins
TIPs	Tonoplast intrinsic proteins
NIPs	NOD26-like intrinsic proteins
SIPs	Small basic intrinsic proteins
XIPs	X-intrinsic proteins
GIPs	GlpF-like intrinsic proteins
HIPs	Intrinsic hybrid proteins
TM	Trans-membranes
ar/R	selectivity filter Aromatic/arginine selectivity filter
HMM	The Hidden Markov model
qRT-PCR	Quantitative real-time RT-PCR
RNA-seq	RNA Sequencing
Semi-qRTRCR	Semi-quantitative RT-PCR
MW	Molecular weight
pI	Isoelectric point
Plas	Plasma membrane
Vacu	Vacuolar membrane
AA	Amino acid
TMD	Transmembrane domain
ORF	Opening reading frame
DcaGADPH	Glyceraldehyde-3-phosphate dehydrogenase
FPKM	The fragment per kilobase of exon per million fragments mapped
