# Supplementary material for: Genome-Wide Identification and Characterization of Aquaporins and Their Role in the Flower Opening Processes in Carnation (Dianthus caryophyllus)
Source: Molecules. 2018 Jul 29;23(8):1895. doi: 10.3390/molecules23081895 (PMC6222698; doi:10.3390/molecules23081895)
Supplement: Supplementary file 1 [file molecules-23-01895-s001.zip › additional file/Figure S1.docx]

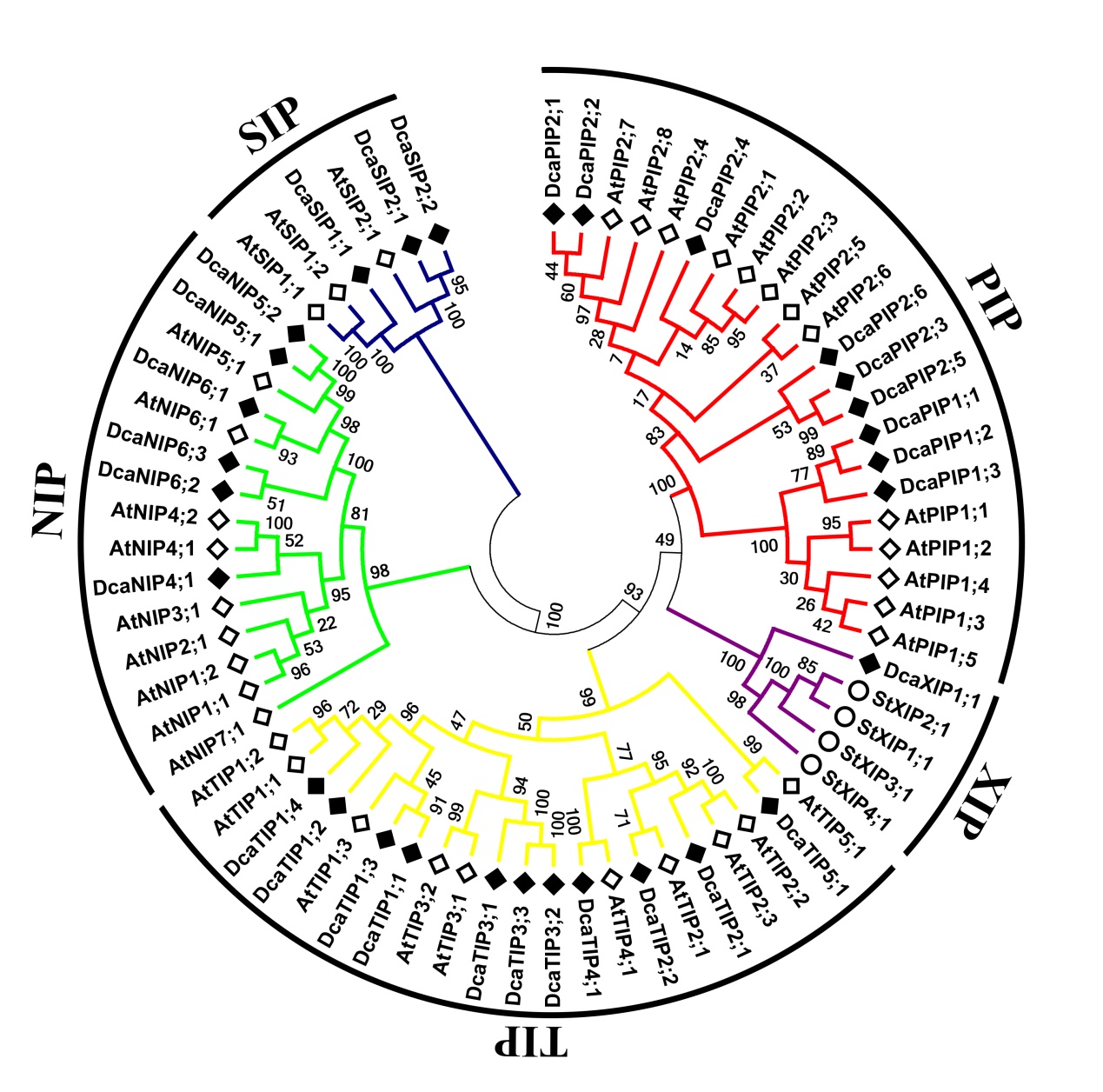


Phylogenetic tree of DcaAQPs with *A. thaliana*, *S. tuberosum*. The different subfamilies were indicted by a circle and different colors. Multiple alignments with other plants species (*A. thaliana*, *S. tuberosum*) by Clustal W and phylogenetic dendrogram by MEGA 6.0 using Maximum Likelihood (ML) method with 1000 bootstrap replicates.
