# Supplementary material for: Genome-Wide Identification and Characterization of Aquaporins and Their Role in the Flower Opening Processes in Carnation (Dianthus caryophyllus)
Source: Molecules. 2018 Jul 29;23(8):1895. doi: 10.3390/molecules23081895 (PMC6222698; doi:10.3390/molecules23081895)
Supplement: Supplementary file 1 [file molecules-23-01895-s001.zip › additional file/Figure S2.docx]

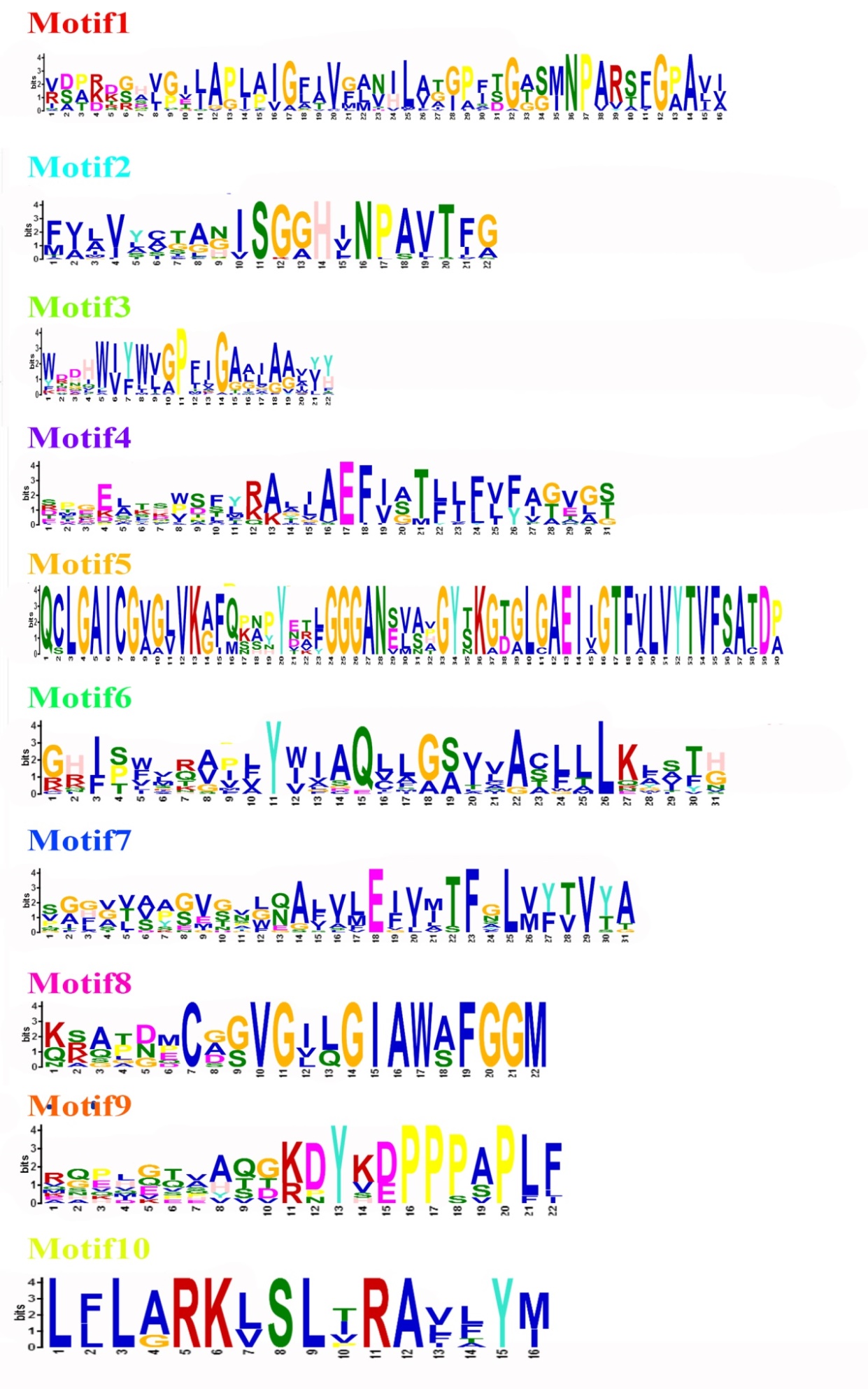


Different motifs are shown by different colors and numbered from 1 to 10 based on MEME suit analysis.
