# Supplementary material for: Genome-Wide Identification and Characterization of Aquaporins and Their Role in the Flower Opening Processes in Carnation (Dianthus caryophyllus)
Source: Molecules. 2018 Jul 29;23(8):1895. doi: 10.3390/molecules23081895 (PMC6222698; doi:10.3390/molecules23081895)
Supplement: Supplementary file 1 [file molecules-23-01895-s001.zip › additional file/Figure S3.docx]

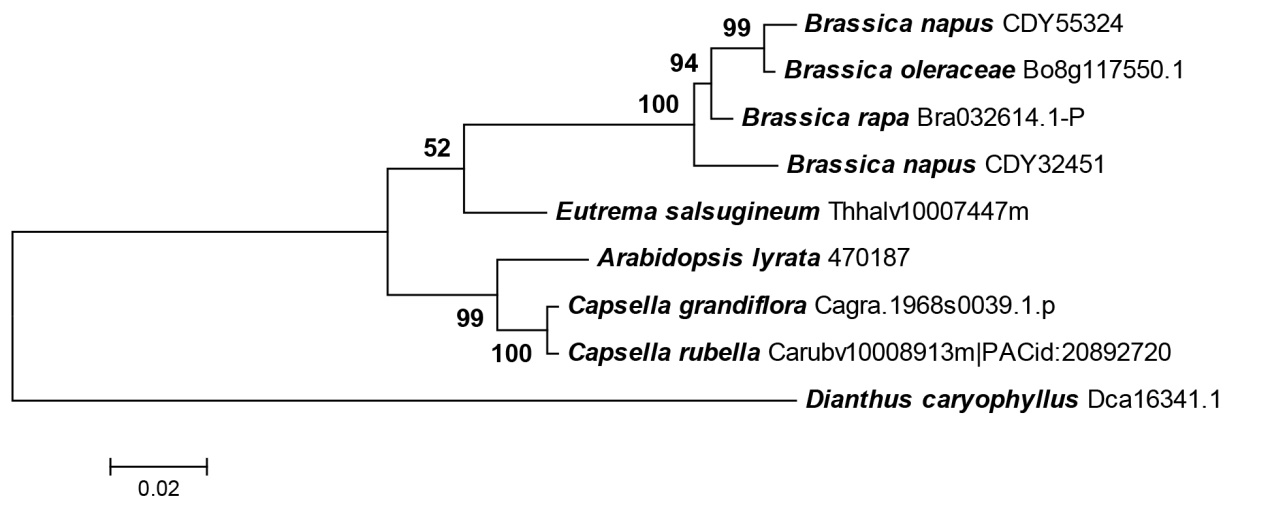


Phylogenetic tree of Si-efflux transporters with Brassicaceae plants (Sonah et al. 2017). Multiple alignments by Clustal W and phylogenetic dendrogram by MEGA 6.0 using Maximum Likelihood (ML) method with 1000 bootstrap replicates.

>Dianthus caryophyllus Dca16341.1

MVMPPTVKVVLGSIAFAIFWVLAVFPAVPFLPIGRTAGSLLGAMLMVLFRVISPEQAYAS

IDLPILGLLFGTMVVSVYLERADMFKYLGKLLAWKSRGPKDLIFRICLISAISSAFFTND

TSCVVLTEFVLKIARQHNLPPHPFLLALASSANIGSSATPIGNPQNLVIAVQSKISFGEF

LIGIVPAMLVGVLVNTLIIIAMYWKLLSTHKDEEDATSEVVDEDDVTSHRFSPATLSHSN

SLEFDSRSLSGVRGHSGHLDTLRNRVIPTENEIQSYESSRNSDASKDVAVNESPEQKSDT

ASVEKVETINGNLHSQLRSFCGGEGLNEKWKQVLWKISVYAVTIGMLIALLLGLNMSWTA

ITAALALVVLDFKDARPSLEKVSYSLLIFFCGMFITVDGFNKTGIPSKFWDLMEPYSRIN

RASGIAVLALVILFLSNVASNVPTVLLLGGQIAASAGMISASEEKKAWLILAWVSTVAGN

LSLLGSAANLIVCEQARRAPHLGYNLTFWRHLKFGVPSTLVVTAIGLTLIRGRMRMTTRP

VGGEEGHDKRSLTGRKGIYGIGYVPYLPRLNTESPIL
