# Supplementary material for: Genome-Wide Identification and Characterization of Aquaporins and Their Role in the Flower Opening Processes in Carnation (Dianthus caryophyllus)
Source: Molecules. 2018 Jul 29;23(8):1895. doi: 10.3390/molecules23081895 (PMC6222698; doi:10.3390/molecules23081895)
Supplement: Supplementary file 1 [file molecules-23-01895-s001.zip › additional file/Supplementary Materials.docx]

Table S1: DcaAQPs sequences were newly identified in this study.

Table S2: All primers were used in this study.

Figure S1: Phylogenetic tree of DcaAQPs with *A. thaliana*, *S. tuberosum*

Table S3: The Ka/Ks ratios of NIP gene pairs in carnation

Figure S2: The information of Motifs 1 to Motifs 10

Table S4: FPKM in three carnation cultivars

Figure S3: Si-efflux transporters sequence in carnation and Phylogenetic tree of Si-efflux transporters with Brassicaceae plants
