# Supplementary material for: Genome-Wide Identification and Characterization of Aquaporins and Their Role in the Flower Opening Processes in Carnation (Dianthus caryophyllus)
Source: Molecules. 2018 Jul 29;23(8):1895. doi: 10.3390/molecules23081895 (PMC6222698; doi:10.3390/molecules23081895)
Supplement: Supplementary file 1 [file molecules-23-01895-s001.zip › additional file/Table S3.docx]

The Ka/Ks ratios of NIP gene pairs in carnation

| No. | Gene pairs | Ks(dS) | Ka（dN） | Ka/Ks（dN/dS） |
| --- | --- | --- | --- | --- |
| 1 | DcNIP4;1/5;1 | 41.7869 | 0.5563 | 0.0133 |
| 2 | DcNIP4;1/5;2 | 41.4883 | 0.5652 | 0.0136 |
| 3 | DcNIP4;1/6;1 | 41.7446 | 0.5277 | 0.0126 |
| 4 | DcNIP4;1/6;2 | 41.9808 | 0.5876 | 0.0140 |
| 5 | DcNIP4;1/6;3 | 41.2951 | 0.5546 | 0.0134 |
| 6 | DcNIP5;1/5;2 | 0.7045 | 0.0644 | 0.0914 |
| 7 | DcNIP5;1/6;1 | 39.5391 | 0.3721 | 0.0094 |
| 8 | DcNIP5;1/6;2 | 6.1620 | 0.3305 | 0.0536 |
| 9 | DcNIP5;1/6;3 | 1.9558 | 0.3465 | 0.1772 |
| 10 | DcNIP5;2/6;1 | 39.7819 | 0.3702 | 0.0093 |
| 11 | DcNIP5;2/6;2 | 5.2729 | 0.3418 | 0.0648 |
| 12 | DcNIP5;2/6;3 | 38.3448 | 0.3244 | 0.0085 |
| 13 | DcNIP6;1/6;2 | 40.2562 | 0.3871 | 0.0096 |
| 14 | DcNIP6;1/6;3 | 5.9239 | 0.3438 | 0.0580 |
| 15 | DcNIP6;2/6;3 | 6.4464 | 0.2751 | 0.0427 |

Non-synonymous (Ka) and synonymous (Ks) substitution ratio of NIP genes were calculate selection pressure. The alignments generated by ClustalW and the corresponding cDNA sequences were submitted to the online program PAL2NAL (<http://www.bork.embl.de/pal2nal/>) (Suyama et al. 2007), which automatically calculates Ks and Ka by the codeml program in PAML (Yang et al. 2007).
